# Pharmaco-fMRI: A Tool to Predict the Response to Antiepileptic Drugs in Epilepsy

**DOI:** 10.3389/fneur.2019.01203

**Published:** 2019-11-13

**Authors:** Fenglai Xiao, Matthias J. Koepp, Dong Zhou

**Affiliations:** ^1^Department of Neurology, West China Hospital of Sichuan University, Chengdu, China; ^2^Department of Clinical and Experimental Epilepsy, UCL Institute of Neurology, London, United Kingdom; ^3^MRI Unit, Epilepsy Society, Chalfont St Peter, United Kingdom

**Keywords:** functional MRI, epilepsy, antiepileptic drugs, pharmaco-fMRI, side-effects, drug response

## Abstract

Pharmacological treatment with antiepileptic medications (AEDs) in epilepsy is associated with a variety of neurocognitive side effects. However, the mechanisms underlying these side effects, and why certain brain anatomies are more affected still remain poorly understood. Advanced functional magnetic resonance imaging (fMRI) methods, such as pharmaco-fMRI, can investigate medication-related effects on brain activities using task and resting state fMRI and showing reproducible activation and deactivation patterns. This methodological approach has been used successfully to complement neuropsychological studies of AEDs. Here we review pharmaco-fMRI studies in people with epilepsy targeting the most-widely prescribed AEDs. Pharmco-fMRI has advanced our understanding of the impact of AEDs on specific brain networks and thus may provide potential biomarkers to move beyond the current “trial and error” approach when commencing anti-epileptic medication.

## Introduction

Epilepsy is one of the most common neurological disorders, characterized by neurobiological, cognitive and psychosocial impairments. Over 20 anti-epileptic medications (AEDs) with various mechanisms of actions are available to suppress seizures, but refractoriness to pharmacological treatment still occurs in approximately 30% of patients with epilepsy (1). After failing two AEDs, the chance of achieving long-term seizure freedom with the addition of any further drugs is <15% no matter what AEDs are used (2). People with pharmaco-resistant epilepsy show remarkably higher trend of neurocognitive comorbidity, morbidity and premature mortality than people with better seizure control. Cognitive impairment in epilepsy, a frequent comorbidity, is due to multiple factors: AEDs, genetic factors, and seizures. Cognitive dysfunction contributes to psychological disturbance, reduced employability and social disadvantage in people with epilepsy (3). Cognitive side effects are often the main complaint of a person with epilepsy, which lead to non-concordance with AED treatment, increasing risk of injury and death. The neural basis of cognitive effects remains unclear and the individual susceptibilities for adverse events are ill-defined. At present, we have no means other than “trial-and-error” to predict the most effective and best-tolerated treatment with AEDs. In particular, there are no reliable predictors for cognitive side-effects.

Advances in neuroimaging techniques contribute to the clinical diagnosis and management of epilepsy (4, 5). Functional magnetic resonance imaging (fMRI) identifies highly reproducible functional brain maps of activation or deactivation patterns triggered by performing specific cognitive tasks such as language, motor and memory (1, 6). This method explores disease-related effects on both localized and network-level fMRI brain maps, and may improve the understanding of specific epileptic syndromes. Disease processes underlying epilepsy implicate complex large-scale brain network interactions beyond the epileptic focus. Recent advances in fMRI methodology allow us to study the impacts of medications and their effects on specific cognitive networks in those who receive neurocognitive pharmacological treatments. This so-called pharmaco-fMRI is a methodology based on the presumption that patterns of activation or deactivation can be affected and quantified in a differential manner by various AEDs (7, 8). Pharmaco-fMRI studies may provide surrogates biomarkers to investigate drug effects at the network-level and to predict the response and cognitive side effects of AEDs. Considering the more than 20 AEDs used in the treatment, and the various functional brain networks involved in the heterogeneity of specific epilepsy syndromes, early determination of AED efficacy and likelihood of neurocognitive adverse effects through fMRI methods are in urgent need.

## Role of Pharmao-fMRI Studies in Epilepsy

Functional MRI has long been used to detect the underlying neurophysiological and anatomic mechanisms of specific behavior and stimuli on various conditions. It measures neuronal activity in an indirect manner via a signal called the Blood-Oxygen-Level-Dependent (BOLD) contrast. This signal derives from changes in the ratio between oxygenated and deoxygenated hemoglobin because of metabolism elicited by neuronal activity. Functional MRI can be scanned when subjects execute specifically designed cognitive tasks, testing for expressive language, episodic and working memory, executive functions and sensory-motor processing (3, 9–12), so-called task-based fMRI. On the other hand, resting-state fMRI techniques detect spontaneous fluctuations of BOLD contrast during “rest,” which means subjects are scanned during task free conditions (4, 13). Resting-state fMRI identifies “functional connectivity” between brain regions, which are a stable reproducible group of cortical and subcortical regions with strongly correlated signal time-courses. These sets of brain regions are also detected in cognitive fMRI with high correspondence with task-implicated systems. In the presurgical evaluation of people with pharmaco-resistant epilepsy, fMRI is clinically utilized to identify brain regions of interest that are crucial for memory, sensori-motor functions and speech. In combination with white matter tractography, MRI methods can reveal white matter tracts which play a vital role in the functions with high correspondence, thus lowering the risk of morbidities inflicted by epilepsy surgery (5). In addition, simultaneous electroencephalography (EEG) and fMRI methodology can be used to help detect and localize the epileptogenic focus and help in planning the implantation of intracranial EEG electrodes (5).

Imaging studies examining the effects of AEDs in epilepsy have been performed mainly with functional MRI methods using non-standardized task or rest-state paradigms, and have been so far cross-sectional (14).

Pharmaco-fMRI studies in epilepsy are challenging for various reasons: (i) patients often need to start treatment immediately with AEDs; (ii) the influences of co-medicatons and other confounders, such as seizure duration or comorbidities have to be considered; (iii) the changes in resting-state fMRI in relation to one specific add-on medication may be too weak to be detected (15, 16). Pharmaco-fMRI studies thus far have been mainly retrospective and cross-sectional (17–20), and mostly used task-based fMRI (18–20). Medications effects are studied as an interactive influence during cognitive tasks fMRI scan (6, 21). The brain maps of activation and deactivation patterns in one specific cognitive fMRI task can be compared for a medication and a placebo condition but the interpretation of the results must be considered within the context of how the illness influences neurovascular coupling (22). Pharmaco-fMRI can explore effects of medications at a highly connected network of brain regions of highest densities of medication targeting effects (23). Thus, pharmaco-fMRI enables to assess large-scale cortical and subcortical systems, providing functional brain maps across different cognitive tasks, irrespective of the different pharmacodynamic properties (24). Pharmaco- fMRI provides mechanism-related activation and deactivation maps which can serve as targets for testing drug effects. A growing number of recent pharmaco-fMRI studies have shed a light on mapping possible mechanisms behind cognitive side effects of AEDs (18–20, 25), corroborating and extending the findings reported in previous neuropsychological studies. AEDs appear to ameliorate either task-related activation or task-relevant deactivation in brain maps including cortical and subcortical areas, which are important for the specific epilepsy syndrome as well as the brain networks responsible for neurocognitive function.

In this review, we performed a pubmed search using the search terms “pharmaco-fMRI,” “epilepsy,” “fMRI,” and “AED.” We only selected manuscripts, which were original articles and includes AED-related functional MRI studies (task and rest-state) in patients with epilepsy. We summarize pharmaco-fMRI studies of the most commonly used AEDs, and contrast these findings in pharmaco-resistant epilepsies to studies of so-called “benign” non-lesional focal epilepsies, which often go untreated.

### Valproate

Valproate (VPA) is a widely used AED used for treating both focal and generalized epilepsy syndromes (26). VPA acts on both, neurotransmitter-dependent and non-dependent cellular conditions. Amongst its many putative mode of actions, it increases GABA turnover rates, thus empowering GABAergic function in the specific set of brain areas. Furthermore, VPA mediates neuronal excitation through a NMDA subtype of glutamate receptors (27). Given the various molecular and cellular patho-mechanisms underlying different seizure syndromes, the diversity of VPA's neurophysiologic and cellular properties of VPA might explain its broad-spectrum antiepileptic efficacy.

**Figure 1:**
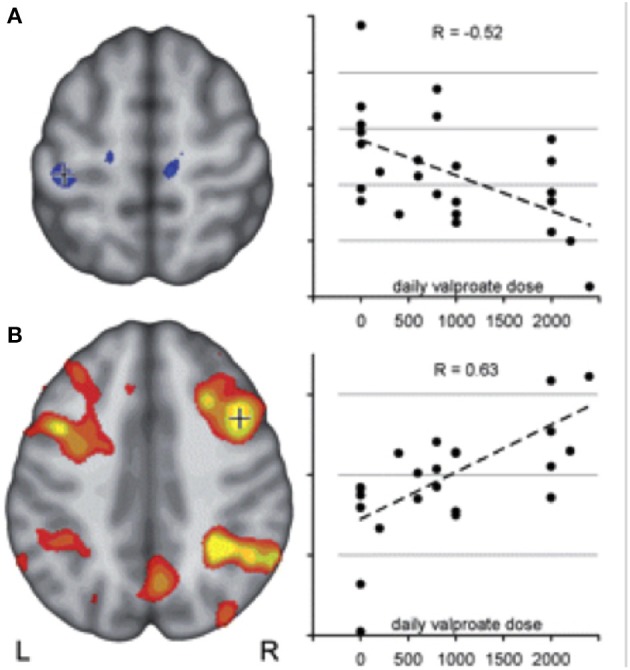
Motor cortex coactivation correlates with valproic acid dosage in JME duting working memory task fMRI. Co-activation of motor and cognitive areas during a visuo-spatial working memory task was detected in JME compared with controls. The figure illustrates: **(A)** a negative correlation between left central activation (voxels underneath crosshair) and daily valproate dose indicating a normalizing effect of VPA on motor cortex hyperactivity. **(B)** Activity within the typical bilateral frontal and parietal working memory network, on the other hand, correlated positively with valproate dose, indicating a normalizing effect of valproate on the cortical activation pattern in JME. Reproduced with permission from Vollmar et al. (17).

VPA is considered as the first-choice AED in juvenile myoclonic epilepsy (JME) (26). Previous imaging analysis revealed that structural as well as functional connectivity are increased among motor and prefrontal brain cognitive networks in JME, likely explaining myoclonic jerks triggered by demanding cognitive activities, a reflex trait known to occur frequently in this syndrome (17, 28). JME patients showed increased activation of the primary motor cortex and supplementary motor area (SMA) during fMRI working memory task with an increasing load of cognitive task, which was modulated by disease factors, including seizure duration and seizure frequency. In addition to co-activation of motor areas, default mode network (DMN) areas failed to de-activate during cognitive tasks (17). Both abnormal co-activation in SMA and impaired deactivation in DMN were attenuated with increasing VPA dose (Figure 1). This is in keeping with the clinical experience that VPA is particularly effective in treating myoclonic jerks in JME without any cognitive side effects (17, 29).

Using interleaved fMRI/transcranial magnetic stimulation (TMS) methodology, TMS was applied to the motor regions in a placebo-controlled, combined fMRI/TMS study. VPA and lamotrigine (LTG) demonstrated different network specific effects: both medications reduced effective connectivity in relation to TMS between the primary motor and prefrontal areas and also between the primary motor and SMA. While TMS was applied over the prefrontal cortex, only LTG was found to be associated with higher effective connectivity between anterior cingulate cortex and the left dorsolateral prefrontal region (30).

In a recent resting state fMRI study of a so-called “benign” type of idiopathic focal epilepsy, rolandic epilepsy (31), measures of regional homogeneity of fMRI time courses were used to investigate the effects of AEDs. Regional homogeneity (ReHo) is a type of measurement of local synchronization of resting-state fMRI time-series across a group of neighboring brain voxels (32). Cortical areas including frontal and centrotemporal regions and subcortical structures like the thalamus, showed attenuation of regional homogeneity in children with VPA treatment compared with untreated children. These regions were believed to be implicated in rolandic/epileptic spike generation in this syndrome. Attenuation of ReHo in centrotemporal regions was found to be dose-related. Compared with levetiracetam (LEV), the VPA effect was more evident in the thalamus but weaker in the cortex. Furthermore, children treated with VPA demonstrated a preserved covariance of functional metrics between thalamus and centrotemporal areas, likely suggesting a balanced effect of VPA on both cortex and subcortical regions (31). These findings support the clinical impression that VPA is the drug of choice for generalized epilepsies with broad efficacy.

In focal epilepsies, structural imaging studies found the use of VPA to be associated with reduced parietal cortical thickness and reduced total gray and white matter volume (33). This effect could possibly be explained by its influence on brain development. Hence, VPA's variable effects require further imaging studies with multi-modality investigations in well-characterized cohorts of patients and healthy controls, which would allow for better understanding of its effects on anatomo-functional trajectories.

### Topiramate

Topiramate (TPM) is widely used in monotherapy as well as an add-on treatment of epilepsy, and is also clinically used as a migraine prophylaxis (34). Cognitive dysfunctions along with TPM use have long been reported in people with epilepsy or migraine and healthy controls, which include impaired short-term memory, reduced sustained attention and decreased psychomotor speed. Impaired working memory and dysfunctional expressive language are frequently reported (35–39). These dysfunctions are even noted after single-dose administration and on steady-state doses in mono- or add-on treatment despite good seizure control (38).

TPM is the most studied AED using pharmaco-fMRI: a total of five studies employed either expressive language tasks after a single dose in healthy subjects or, in people with either epilepsy or migraine, on a steady dose of TPM. Given the importance that effective deactivation of the DMN has played an equally important role in executing task successfully in cognitive fMRI scans (40), these studies suggest an underlying mechanism by which TPM impairs cognitive processing during speech function. They shared the following functional abnormalities: (i) Activation was reduced in brain areas relevant for language, including inferior frontal and middle frontal gyri (IFG and MFG), superior temporal gyrus in the language-dominant hemisphere (41–43). (ii) DMN regions failed to deactivate in subjects who executed language task (25, 43, 44). In addition, activation during verbal fluency task correlated with TPM dosages in the precuneus (25), which is an essential part of the DMN (21) and also plays a part in language networks when expressive language functions are engaged (45). More importantly, similar cognitive side effects and disturbance of language task relevant DMN deactivations were also described in healthy controls with the use of TPM (25). Overall, the above fMRI findings highlight the sensitivity of pharmaco-fMRI to detect the neurocognitive side effects of AEDs on functional brain networks.

### Zonisamide

**Figure 2:**
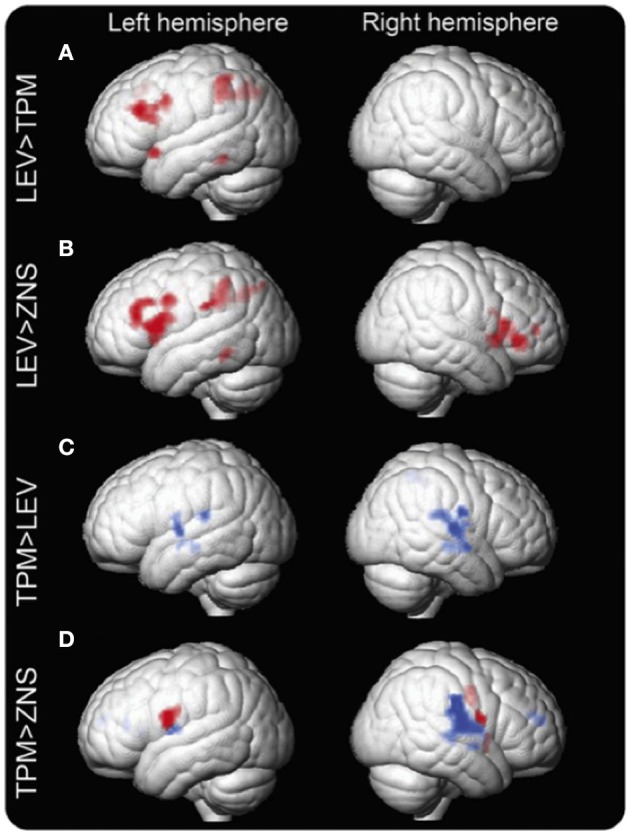
Group differences in the verbal fluency task fMRI activation maps between LEV, TPM, and ZNS. Group differences in fMRI activation maps during the verbal fluency task Significant group differences between patients on levetiracetam (LEV), topiramate (TPM), and zonisamide (ZNS) are demonstrated. Patients on TPM and ZNS have less activation in frontal and parietal cognitive networks than patients on LEV. In patients on TPM, activation is reduced in the left middle frontal gyrus (MFG) and left dorsal parietal region **(A)**. In patients on ZNS, activation is reduced in the left MFG and bilateral inferior frontal gyrus (IFG), as well as the left dorsal parietal region **(B)**. In terms of task-relevant deactivation networks, bilateral lateral temporal regions and rolandic opercula and the right inferior parietal lobule and supramarginal gyrus are less deactivated (blue) in patients on TPM compared to those on LEV **(C)**. Compared to ZNS, TPM shows increased activation in the IFG, insular cortex, and rolandic operculum on the left and the insular cortex, inferior parietal lobule, supramarginal gyrus, superior temporal gyrus, and rolandic operculum on the right. Differences in the left are due mainly to increased activation of task-relevant regions as shown in red (inclusively masked with LEV activation maps); on the right, activated regions lie mainly within task-negative areas, i.e., are due to impaired deactivation as shown in blue (inclusively masked with LEV and ZNS deactivation maps) **(D)**. *p* < 0.005, 20-voxel threshold extent. Reproduced from Wandschneider et al. (19).

Zonisamide (ZNS) is used to treat both focal and generalized epilepsies, and leads to neurocognitive side effects similar to TPM albeit more moderate (39). Mechanisms of drug action of ZNS include modulation of dopaminergic and serotoninergic transmission, blockade of voltage-sensitive sodium channels and T-type calcium channels, as well as a neuroprotective effect from free-radical damage (46). One recent retrospective study of verbal fluency fMRI compared people with focal epilepsy syndromes taking TPM, ZNS, and LEV. Wandschneider et al. described a similar drug effect of ZNS and TPM on frontoparietal cognitive networks (19) (Figure 2). However, altered deactivations in the DMN including lateral temporal regions and inferior parietal lobes were found in people treated with TPM but not in the ZNS group. Activations of parietal structures, which support general task performance in the cognitive tasks including working memory and sustained attention system, in addition to activation of frontal networks relevant for language becomes apparent with the increase of cognitive demand in this study (47). Considering the execution of the verbal fluency task requires relatively low cognitive demand, decreased activation in expressive language-specific regions (IFG and MFG), sustained attention (parietal cortex) and working memory (frontoparietal lobes) implies that TPM and ZNS might suppress higher-level neurocognitive processing.

### Carbamazepine

Carbamazepine (CBZ) was the first AED to be investigated in a pharmaco-fMRI design in patients with epilepsy (48). Twenty-one people with pharmaco-resistant temporal lobe epilepsy (TLE) performed a visuo-spatial memory retrieval task, which elicited activations of mesiotemporal regions by the means of cognitive navigation through a familiar route in the scanner. They observed reduced brain activations within mesiotemporal areas with the increased CBZ serum levels, independent of the lateralization of the epileptic foci (48).

### Oxcarbazepine

One recent pharmaco-fMRI study in people with TLE using resting-state fMRI compared a subgroup of participants treated with CBZ or oxcarbazepine (OXC) with people with other AEDs. Using a graph-theoretical approach to characterize the organizational properties of functional networks, abnormal “hubness” was reported in people treated with CBZ/OXC (49): while less highly connected “hubness” was detected in the CBZ/OXC group, “betweenness centrality” was decreased in the thalamus and limbic circuit, but increased in the DMN regions including cingulate and posterior cingulate/precuneus. These results suggest redistribution of “hubness” with more remarkable shifts from limbic to lateral cortices in people with TLE on CBZ/OXC. Previous fMRI findings in TLE with graph theoretical analysis showed a “re-distribution” of hubness areas with high betweenness centrality to mainly temporal association cortical areas and paralimbic and (50). Hence, the findings in this study suggest a region-specific drug effect of CBZ/OXC on epilepsy-related brain network changes. Another interesting finding in this study is that attenuation of activation of mesiotemporal lobes was dose-dependent.

### Levetiracetam

Levetiracetam (LEV) is one of the most widely used AEDs for focal and generalized epilepsies with good efficacy and tolerance (51). LEV binds to the synaptic vesicle protein SV2A. Its mechanism is believed to be via the modulation of synaptic neurotransmitter release (52). In most neuropsychological studies LEV has been shown to have a favorable cognitive profile (53), exerting a positive impact on cognition, and as a result even ameliorate neurocognitive performance (54, 55).

In keeping with these observations, pharmaco-fMRI studies showed a beneficial impacts of LEV on neurocognitive networks (18–20). One recent pharmaco-fMRI study compared people on LEV with those not treated with LEV via a verbal and visual-spatial working memory task in people with unilateral TLE. People on LEV showed more extended DMN deactivated regions relevant for the task in the affected temporal lobe than people not treated with LEV. Specifically, this effect was observed (i) in the left mid-temporal gyrus in people with left TLE during the verbal task; (ii) in the right hippocampus in those with right TLE performing the visual- spatial task. These drug effects became more obvious with increasing LEV dosages, suggesting a significant dose-dependence. This study revealed the task-specific difference of effects on syndrome-specific fMRI regions between left and right TLE. Since people taking LEV showed similar task-related brain maps of activation and deactivation patterns with healthy controls, LEV is believed to be associated with normalizing effects on task fMRI brain activation and deactivation patterns in people with epilepsy (18).

Previous fMRI studies investigating the functional networks of working memory illustrated that amelioration of activation of mesiotemporal lobes contributed to effective task performance (56, 57). This may occur as part of a brain activity resource redistribution from task-irrelevant to task-relevant cortices in order to reduce interference (58). Recent cognitive fMRI studies examining working memory in people with TLE described failure to deactivate the ipsilateral hippocampus to the presumed epileptic focus in comparison with healthy controls. This kind of derangement points to a disruption in the segregation between task-negative and task-positive regions, specifically mesiotemporal and parietal lobes (59, 60).

In children with rolandic epilepsy, this effect of LEV was also observed in resting-state fMRI: in comparison with drug-naïve children, lower ReHo was found in children on LEV in frontal and centrotemporal cortices and subcortical areas including thalamus and basal ganglia. These regions are believed to be involved in the generation of rolandic spikes. Comparing ReHo patterns in children treated LEV with subjects on VPA revealed different spatial specificity of the effects of these two AEDs. Specifically, LEV had a pronounced effect on frontal and temporal regions and caudate while exerting a less evident impact on thalamus. Additionally, LEV had dissociating effects on the fMRI local covariance metrics of thalamus and centrotemporal regions (31). However, the absence of healthy controls in this study does not allow us to establish whether ReHo patterns in children on LEV may reflect a “normalizing effect” to normal baseline status.

People with amnestic mild cognitive impairment who have a risk of progressing into Alzheimer's disease were studied with pharmaco-fMRI and LEV. Abnormally higher activation in hippocampus in the dentate gyrus/CA3 regions was attenuated with the administration of low-dose LEV which was corroborated by improvement of out-of-scanner memory measures (61, 62).

Overall, pharmaco-fMRI revealed specific effects of LEV with predominant focus on the networks in relation to pathomechanisms underlying diseases. Its effects of restoring abnormal activation and deactivation patterns may explain its positive cognitive profile.

### Lamotrigine

Lamotrigine (LTG) is effective for both focal and generalized epilepsy syndromes, which are recorded to be associated with fewer cognitive and behavioral changes compared with other AEDs (63, 64). In a retrospective study using verbal fluency fMRI, we investigated the cognitive side effects of sodium channel–blocking AEDs in people who had been taking CBZ or LTG, while we also included healthy controls (20). Forty-two people on levetiracetam (LEV) were used as patient controls because of LEV's “normalizing effects” on cognitive activation patterns (18). In people treated with LTG, abnormal fMRI findings were limited to failed deactivation of the DMN regions, while those on CBZ showed decreased activations in the dominant IFG. Clinical measures of category and word fluency tests found that the performance of people treated with CBZ was worse than those treated with LEV or LTG.

### Combination Studies

**Figure 3:**
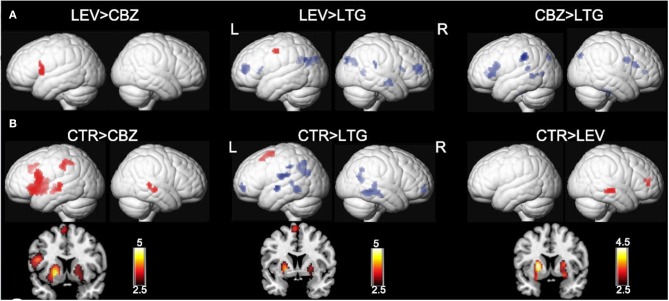
Effects of carbamazepine and lamotrigine on functional magnetic resonance imaging cognitive networks. Significant group differences between patients on carbamazepine (CBZ), levetiracetam (LEV), and lamotrigine (LTG) and healthy controls (CTR) are demonstrated on a surface-rendered brain template, and the subcortical changes are demonstrated on a coronal brain template with a bar chart indicating z score. **(A)** Patients on CBZ activated less in the left inferior frontal gyrus than patients on LEV. In patients on LTG, deactivation was reduced in the task-negative areas, including the middle frontal gyri and left dorsal parietal region of the precuneus, compared with patients on LEV and CBZ. *P* < 0.005, 10-voxel threshold. **(B)** All three groups of patients activated the putamen less bilaterally (left > right) than CTR. Patients on CBZ activated the left inferior frontal gyrus less than CTR. There was less deactivation in the bilateral medial frontal gyri and bilateral lateral temporal regions, left rolandic operculum, and left inferior parietal lobule in patients on LTG. *P* < 0.005, 10-voxel threshold. L, left; R, right. Reproduced with permission from Xiao et al. (20).

The combination of these two pharmaco-fMRI studies provided preliminary evidence for altered activation of task-related regions and failed deactivation in the DMN (Figures 2, 3), which is highly correlated with progressively worse neurocognitive side effects (Table 1):

- LTG usage is associated only with abnormal deactivation in the DMN, which has a limited impact on clinical language performance;
- ZNS and CBZ show similar dysfunctions in the activation patterns relevant for language, and both medications are associated with poorer performance in verbal fluency;
- TPM is associated with worse cognitive functions compared with other AEDs, and results in both, decreased language- specific frontal fMRI activations and altered task-relevant deactivation of the DMN regions (19, 65).

**Table 1:** The summary of impaired activation and deactivation in verbal fluency task pharmaco-fMRI studies in epilepsy.

**AEDs**	**Activation**	**Deactivation**	**Effects on cognition**
Topiramate	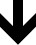	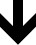	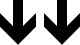
Zonisamide	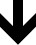	N/A	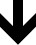
Carbamazepine	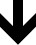	N/A	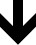
Lamotrigine	N/A	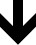	N/A

*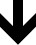, impaired; 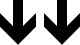, severely impaired*.

## Neuroimaging in Drug-Sensitive Epilepsies

Functional imaging studies may help to phenotype individuals who show positive responses to certain AEDs or distinguishing responders from non-responders. In one resting state fMRI study with genetic generalized epilepsy, Szaflarski et al. compared people who are responsive to VPA and those who are not. Their findings suggest that people with VPA-responsive and VPA-resistant genetic generalized epilepsy might have different generalized spike waves discharges (GSWD) generators, and that these differences in GSWD generators are likely to account for the different responses to VPA. Furthermore, reduced functional connectivity was more prominent in treatment-resistant epilepsy and functional connectivity was significantly reduced with greater duration of epilepsy (66).

One recent study in rolandic epilepsy explored the difference in dysfunctional white matter networks between treated and untreated children (67). Based on previous findings of white matter abnormalities in the rolandic networks (68, 69), Jiang et al. obtained several white-matter functional networks and analyzed conventional functional connectivity in four frequency sub-bands. They further employed functional covariance connectivity (FCC), which is an indicator for the estimation of the covariance between two white matter networks based on their correlations with multiple gray matter regions (70, 71). In comparison with healthy children, the untreated group showed increased functional connectivity and FCC in rolandic regions and sensorimotor network (precentral and postcentral regions), and decreased functional connectivity and FCC in dorsal frontal network while these abnormalities were not evident in the those children with AED treatment. These results are in consistence with our previous fMRI findings showing underlying functional network abnormalities in this “benign” type of epilepsy, which appear to be responsive to AED treatment (72). Methodological approaches of combined functional and structural MRI metrics analysis offer the promising outcome predictors for the future selection of suitable AED treatment choices with efficacy as well as good tolerance.

## Conclusion

The neuroanatomical mechanisms underlying the drug effects of AEDs are more complex than just a general decrease in cortical neuronal excitability (73, 74). Seizure control measures often rely on subjective ratings, and as such seizure frequency according to the patients' or care-givers' accounts are often unreliable. Neuroimaging techniques, particularly fMRI, have largely contributed to clinical presurgical evaluation in epilepsy treatment, but also have the potential to provide further insights into the mechanisms underlying effects of AEDs. Pharmaco-fMRI studies investigate AEDs' effects on functional brain networks, and could help to determine early treatment response and unravel mechanisms of drug efficacy and adverse effects, and through this advance new AED research and development. One limitation of current pharmaco-fMRI studies in epilepsy is the variability of techniques used and a lack of standard for methodology (22). The challenges faced in methodology of pharmaco-fMRI could be mitigated by choosing suitable experimental drug and matching drug effects to regional BOLD signal (22). In addition, study design with multimodal complementary approaches including ASL, electrophysiological measurements and pharmacogenetics could also be utilized to provide additional information of the complex patterns of the brain's functional anatomy.

For now, most pharmaco-fMRI studies in epilepsy either cross-sectional or retrospective. Longitudinal studies will be required, in which causality can be addressed, controlling for the influences of clinical, psychosocial, and medication-related factors. Ideally, pharmaco-fMRI studies should recruit people who are going to have AED treatment at different time intervals from new-onset to chronic disease throughout their disease course, with a complete battery of neuropsychological testing. Specifically, prospective studies of people with new-onset epilepsy enables to better prevent medication-related effects from the contamination of epilepsy-related cofounders.

Current pharmaco-fMRI studies in epilepsy provide spatially detailed information at the group level. If AED-related fMRI effects can be utilized for individually precision medicine remains to be shown. Jirsa et al. proposed a “Vitual Epileptic Patient” to develop individualized whole brain networks for epilepsy, which could be used for future personalized pharmaco-fMRI studies (75).

## Author Contributions

FX: articles search and drafting/revising the manuscript. DZ: drafting/revising the manuscript, study concept, and study supervision. MK: drafting/revising the manuscript, study concept or design, and study supervision.

### Conflict of Interest

The authors declare that the research was conducted in the absence of any commercial or financial relationships that could be construed as a potential conflict of interest.
